# Identification of CD4^+^ Conventional T Cells-Related lncRNA Signature to Improve the Prediction of Prognosis and Immunotherapy Response in Breast Cancer

**DOI:** 10.3389/fimmu.2022.880769

**Published:** 2022-05-04

**Authors:** Shipeng Ning, Jianbin Wu, You Pan, Kun Qiao, Lei Li, Qinghua Huang

**Affiliations:** ^1^ Department of Breast Surgery, Guangxi Medical University Cancer Hospital, Nanning, China; ^2^ Fujian Maternity and Child Health Hospital, College of Clinical Medicine for Obstetrics & Gynecology and Pediatrics, Fujian Medical University, Fujian, China; ^3^ Department of Breast Surgery, Harbin Medical University Cancer Hospital, Harbin, China; ^4^ Department of Pathology, Dunedin School of Medicine, University of Otago, Dunedin, New Zealand

**Keywords:** long non-coding RNA, CD4^+^ conventional T cells, breast cancer, The Cancer Genome Atlas, prognostic signature

## Abstract

**Background:**

Breast cancer (BC) is one of the most common malignancies in women, and long non-coding RNAs (lncRNAs) are key regulators of its development. T cells can recognize and kill cancer cells, and CD4^+^ T conventional (Tconv) cells are the main orchestrators of cancer immune function. However, research on CD4^+^ Tconv-related lncRNAs (CD4TLAs) prognostic signature in patients with BC is still lacking.

**Method:**

A TCGA database and a GEO database were used to collect the BC patients. Through LASSO Cox regression analysis CD4TLAs-related prognostic models were further constructed, and risk scores (RS) were generated and developed a nomogram based on CD4TLAs. The accuracy of this model was validated in randomized cohorts and different clinical subgroups. Gene set enrichment analysis (GSEA) was used to explore potential signature-based functions. The role of RS has been further explored in the tumor microenvironment (TME), immunotherapy, and chemotherapy.

**Result:**

A prognostic model based on 16 CD4TLAs was identified. High-RS was significantly associated with a poorer prognosis. RS was shown to be an independent prognostic indicator in BC patients. The low-RS group had a significant expression of immune infiltrating cells and significantly enriched immune-related functional pathways. In addition, the results of immunotherapy prediction indicated that patients with low-RS were more sensitive to immunotherapy.

**Conclusions:**

Our signature has potential predictive value for BC prognosis and immunotherapy response. The findings of this work have greatly increased our understanding of CD4TLA in BC.

## Background

Breast cancer (BC) is the leading cause of cancer-related deaths and one of the most common cancer in women around the world (1, 2). In 2020, the International Agency for Research on Cancer of the World Health Organization announced that new breast cancer diagnoses surpassed lung cancer diagnoses for the first time. Research on the prevention and treatment of breast cancer remains a crucial concern around the globe. BC is currently treated primarily by surgery and or by combining multiple adjuvant therapies, such as chemotherapy, immunotherapy, targeted therapy, and radiation therapy (3–5). Although early detection and treatment of BC has improved, patient outcomes still remain unfavorable. Therefore, there is a need to intensify the research for novel high-efficiency molecular biomarkers for the treatment of BC patients, and the development of new prognostic signatures must be intensified.

Long non-coding RNAs (lncRNAs) are those with over 200 nucleotides in length (6, 7). In addition to coding for proteins, they participate directly or indirectly in the regulation of numerous life activities. Recent studies suggest that lncRNAs can play a role in innate and adaptive immunity in cancer by mediating the functional status of immune cells, pathways, and genes (8, 9). Findings of Dongdi et al. revealed that lncRNA HOTAIR could affect cell growth, migration, invasion, and apoptosis in BC through the miR-20a-5p/HMGA2 axis (10). LncRNA PCAT7 can promote the malignant progression of BC by regulating the ErbB/PI3K/Akt pathway (11). The lncRNA HAL plays an influential role in regulating the BC microenvironment as well as promoting BC stemness (12). These studies suggest that lncRNAs can be valuable biomarkers and therapeutic targets for treating BC. Recently, progress in immunology have led to the identification of new mechanisms of immune-related disease. Cellular immune responses in tumors are often attributed to CD8^+^ T cells. However, CD4^+^ T cells also play a key role. CD4^+^ T cells can recognize tumor antigens, supporting the initiation and expansion of CD8^+^ T cells in lymphoid tissues (13–15). Matthew et al. showed that CD4^+^ T cells at the tumor site and throughout the body are a key component of immune-mediated tumor rejection (16). As an indispensable part of the immune composition, CD4^+^ T conventional (Tconv) cells play a crucial role in BC. Nevertheless, the application of CD4^+^ Tconv-related lncRNA (CD4TLA) combination in prognosis prediction of BC patients has not been established.

Researchers have been able to study BC in greater depth thanks to the recent development of single-cell sequencing technology. The leading goal of this study was to construct a CD4TLA signature that could be used to predict the prognosis and immunotherapy of BC patients, and possibly insight into clinical treatment options. Based on single-cell data, we screened CD4^+^ Tconv-related genes in BC and, using bioinformatics, constructed a prognostic signature based on CD4TLAs for BC. This study examined the clinical characteristics, prognostic value, and tumor microenvironment (TME) of the obtained risk score (RS) in BC. Furthermore, we tested the efficacy of RS in BC immunotherapy and chemotherapy.

## Materials and Methods

### Breast Cancer Data Source and Preprocessing

The gene expression data and clinical annotations for BC samples (TCGA-BRCA, n=1109) were obtained from The Cancer Genome Atlas (TCGA, https://portal.gdc.cancer.gov/). Patients without survival information were excluded. Fragments per kilobase million (FPKM) were transformed into transcripts per million (TPM) values in the TCGA-BRCA cohort. Data on BC single cells RNA-seq (GSE110686 and GSE11472) were derived from GEO databases (https://www.ncbi.nlm.nih.gov/geo/) (17, 18).

### Identifying CD4^+^ Tconv-Related Differential Expressed lncRNAs Breast Cancer

Tumor Immune Single-cell Hub (TISCH) is a scRNA-seq database which focus on TME (19). To identify CD4TLAs, we first extracted CD4^+^ Tconv-related genes from BC single-cell data through the TISCH database. The correlation between lncRNAs and CD4^+^ Tconv-related genes was calculated using the Pearson correlation. The lncRNA with an absolute |correlation coefficient| >0.5 and a *P* < 0.001 was considered as a CD4TLA. Through the R package “limma”, CD4TLAs that are significantly different (*P* < 0.05) between normal tissue and tumor tissue were identified (20, 21).

### Construction of a CD4+ Tconv-Related lncRNA Risk Scores Model

To screen lncRNAs associated with OS in BC patients, we performed Univariate Cox regression analysis on the significantly different CD4TLAs. These were then subjected to LASSO regression analysis to identify more meaningful prognostic lncRNAs. Finally, 16 CD4TLAs significantly associated with OS were identified, and the risk score (RS) of each patient was calculated based on the expression level of CD4TLAs and the Cox regression coefficient. Patients were divided into high- and low-risk subgroups by median risk score. The ROC curve was used to check the accuracy of the model. In addition, we validated the model by randomly assigning BC patients to validation group 1 and validation group 2. Univariate and multivariate Cox regression analyses examined whether RS could be an independent prognostic factor in BC patients.

### Functional Enrichment Analysis

The Metascape database is a resource for gene annotation and analysis (22). In this study, the Gene Ontology (GO) and Kyoto Encyclopedia of Genes and Genomes (KEGG) pathway enrichment analyses of CD4^+^ Tconv-related genes were conducted using the Metascape database. We performed GSVA enrichment analysis using “GSVA” R package in order to explore the differences in biological processes between high- and low-risk groups. The R package “cluster profile” was used for functional annotation and the gene set file (c2.cp.kegg.v7.2.symbols.gmt) was obtained from the MSigDB database (https://www.gsea-msigdb.org).

### Tumor Microenvironment Analysis

The “estimate” package was used to estimate the composition of the immune stroma in the BC tumor microenvironment (TME) and to derive immune scores, stroma scores, and estimate scores. The ssGSEA algorithm was used to quantify differences in immune cell infiltration subsets and immunological functional enrichment between high- and low-RS subgroups.

### Evaluation of the Immunotherapy Response and Chemotherapy Based on RS

To further evaluate the potential application of RS in clinical treatment, we predicted the response of high- and low-RS subgroups to immunotherapy by the TIDE algorithm (23). Immunotherapy data from patients were obtained from the Cancer Immuneome Database (TCIA), and we further assessed Immunophenoscore (IPS) in high- and low-RS subgroups to predict patient response to immunotherapy (24). In addition, in order to explore the sensitivity of high- and low-RS patients to different chemotherapeutic drugs, we predicted the IC50 of chemotherapeutic drugs by the “pRRophetic” package.

### Statistical Analysis

All statistical analyses were carried out utilizing the R version 4.1.2 and SPSS 23.0. The student’s t-test (unpaired, two-tailed) was used to evaluate the differences between the two independent groups. One-way analysis of variance (ANOVA) and Kruskal–Wallis test were used as parametric and non-parametric methods, respectively, for data from more than two groups. The R packages “survival” and “survminer” were used for survival analysis. Volcano and heatmaps were drawn by the “ggplots” package. *P* < 0.05 was deemed to be significant.

## Results

### Single-Cell Data Analysis of BC Patients

**Figure 1:**
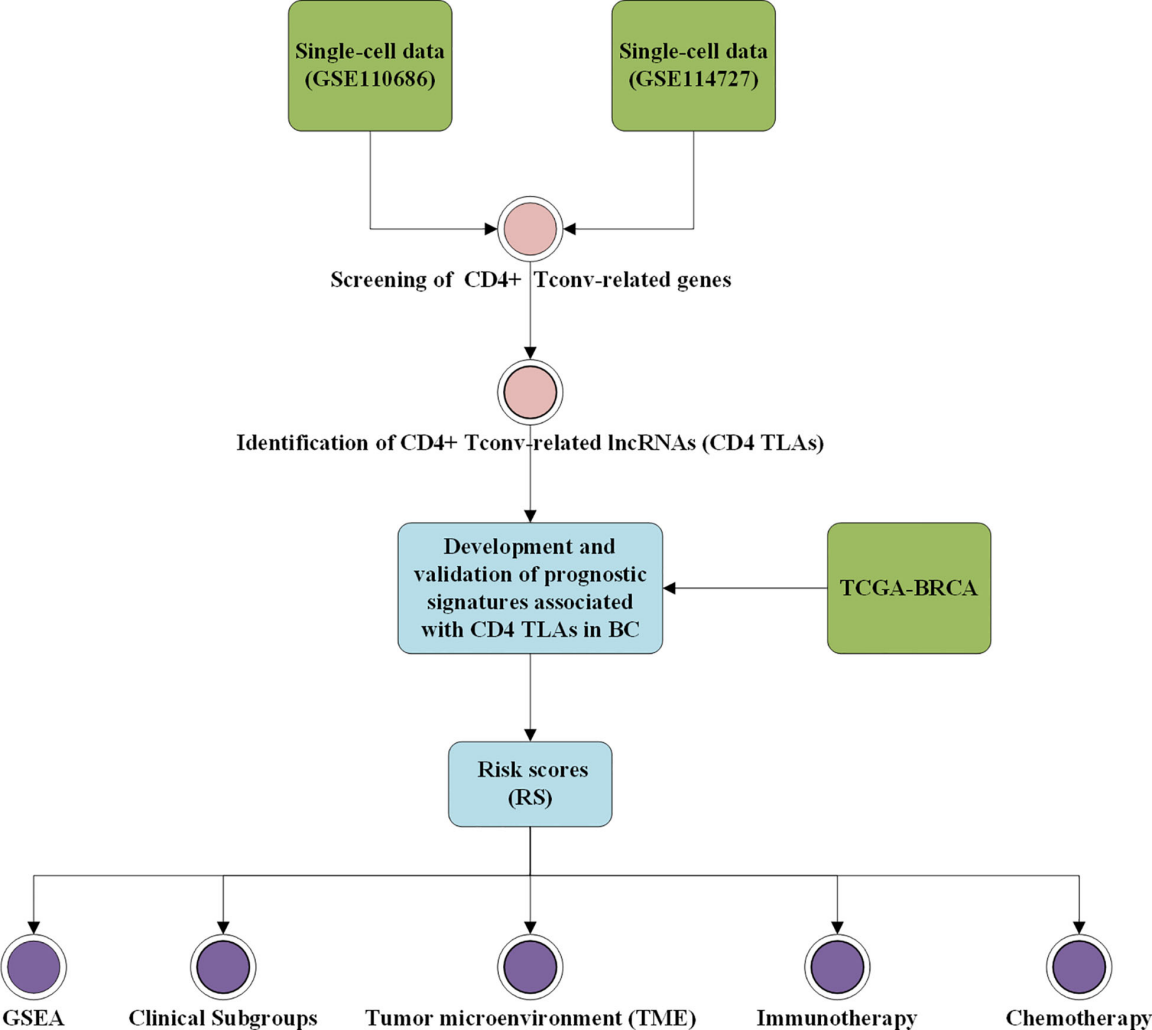
Workflow diagram. The specific workflow graph of data analysis.

**Figure 2:**
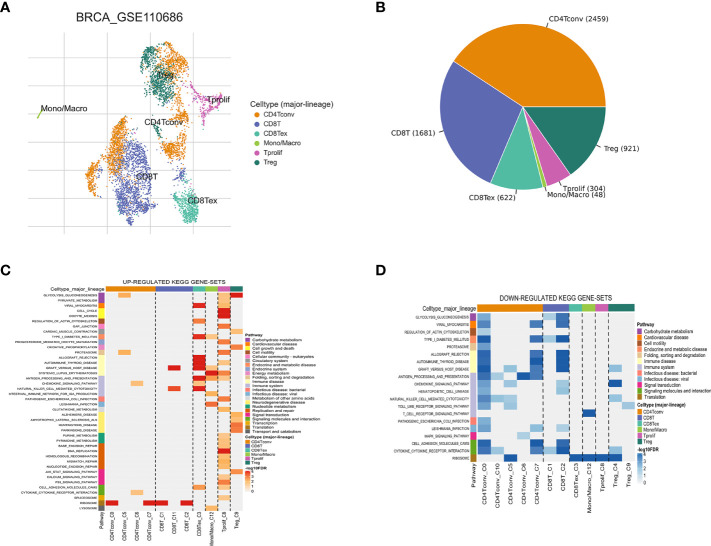
Breast cancer single-cell data analysis based on the GSE110686 dataset. **(A)** UMAP plots with cells colored by cell type are displayed. **(B)** The pie plot shows the cell number distribution of each cell type. Heatmap showing enriched up- **(C)** or down-regulated **(D)** pathways identified based on differential genes in each cell type.

**Figure 3:**
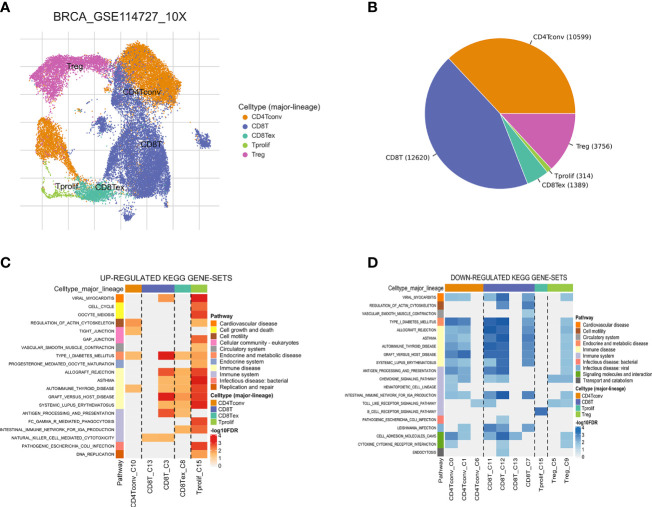
Breast cancer single-cell data analysis based on the GSE114727 dataset. **(A)** UMAP plots with cells colored by cell type are displayed. **(B)** The pie plot shows the cell number distribution of each cell type. Heatmap showing enriched up- **(C)** or down-regulated **(D)** pathways identified based on differential genes in each cell type.

The workflow of this study is shown in **Figure 1**. Based on the TISCH database, we analyzed the GSE110686 and GSE114727 datasets, and compared and visualized the major cell types in their microenvironments (**Figures 2A**, **3A**). We found that CD4^+^ Tconv was the largest subpopulation of cells (**Figures 2B**, **3B**). Furthermore, we analyzed functional differences between different cell types by GSEA enrichment (**Figures 2C, D**, **3C, D**). Findings revealed that CD4^+^ Tconv was significantly enriched in carbohydrate metabolism, signal transduction, translation, signaling molecules and interaction, endocrine and metabolic disease, immune disease, cardiovascular disease, immune system, and cell motility-related pathways. These results suggest that CD4^+^ Tconv has an important role in BC and deserves further study.

### Identification and Screening of CD4^+^ Tconv -Related Genes and lncRNAs in BC

**Figure 4:**
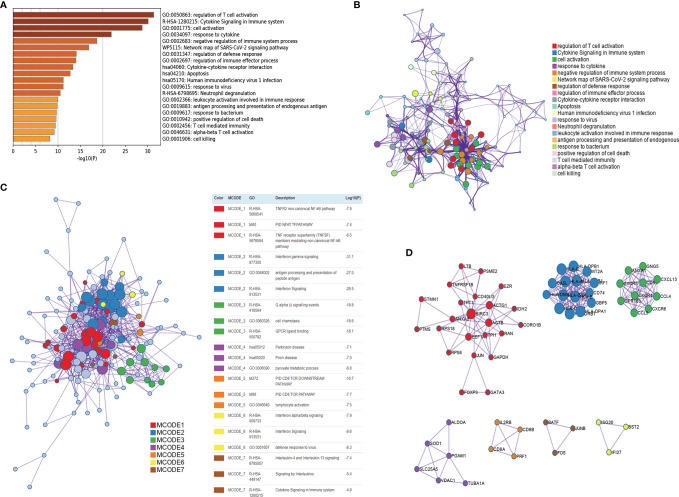
Functional enrichment of CD4^+^ Tconv-related genes and visualization of interactome analysis results. **(A)** Metascape enrichment analysis for the CD4^+^ Tconv-related genes. **(B)** Metascape enrichment network visualization showing the intra-cluster and inter-cluster similarities of enriched terms. **(C)** Metascape visualization of the interactome network formed by CD4^+^ Tconv-related genes candidates, where the MCODE complexes are colored according to their identities. **(D)** Seven MCODE complexes automatically identified in Metascape, colored by their identities. Their functional labels are generated based on the top-three functional enriched terms.

**Figure 5:**
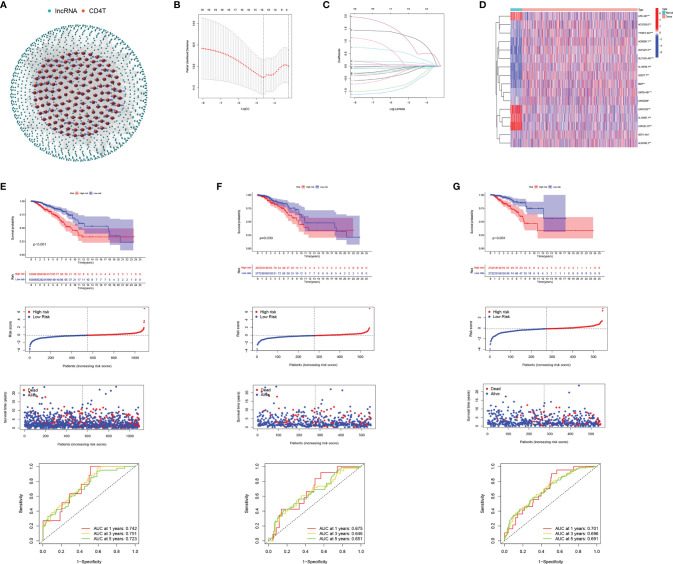
Development and validation of CD4TLAs-Related Risk Signature. **(A)** Co-expression network of lncRNAs and CD4^+^ Tconv-related genes. **(B)** Cross‐validation for tuning parameter selection in the lasso regression. **(C)** Validation was performed for tuning parameter selection through the least absolute shrinkage and selection operator (LASSO) regression model for overall survival (OS). **(D)** The heatmap shows the expression distribution of A. **(E)** CD4TLAs-related prognostic model constructed in total BC patients. **(F)** Validation cohort 1. **(G)** Validation cohort 2. From top to bottom: Kaplan-Meier survival analysis of high- and low-RS subgroups; risk score between high- and low-risk groups; patient survival time; plots of the AUC for time-dependent ROC performance.

The CD4^+^ Tconv-related genes were first extracted from the GSE110686 and GSE114727 datasets and then intersected, yielding 192 CD4+ Tconv-related genes (**Supplementary Figure 1**; **Supplementary Table 1**). The results of functional enrichment showed that they were mainly enriched in immune-related pathways, such as regulation of T cell activation, Cytokine Signaling in the Immune system, negative regulation of immune system process, regulation of immune effector process (**Figure 4A**). To further explore the relationship between enriched pathways, we present it as a network graph, where terms with similarity > 0.3 are connected by edges (**Figure 4B**). In addition, we further performed protein-protein interaction enrichment analysis, which is mainly related to the regulation of cytokine signaling in the Immune system, cell activation, regulation of T cell activation (**Figure 4C**). The MCODE network is shown in **Figure 4D**. A CD4^+^ Tconv-related gene lncRNA (CD4TLA) coexpression network was constructed to identify CD4TLAs (**Figure 5A**). They were then subjected to differential analysis, and finally, 244 CD4TLAs that were significantly different between tumor and normal groups were identified (**Supplementary Figures 2A, B**; **Supplementary Table 2**).

### Development and Validation of Prognostic Signatures Associated With CD4 TLAs in BC

From single-cell data from BC patients, we obtained CD4^+^ Tconv-related genes and identified CD4TLAs that were significantly dysregulated in BC. To explore the role of these CD4TLAs in BC, we further analyzed them based on TCGA-BRCA data. By using univariate COX analysis on the significantly different CD4TLAs, we screened lncRNAs significantly associated with prognosis. Further, we constructed a BC prognostic model by suing the LASSO algorithm and generated a risk score (RS). We finally identified risk signatures of 16 lncRNAs (**Figures 5B, C**). The risk score was calculated as follows: RS = (-0.145 *  AC137630.3 exp.) + (-0.041 *  SIDT1-AS1 exp.) + (-0.510 * AL133467.1 exp.) + (-0.595 *  LIPE-AS1 exp.) + (0.369 * AC012213.3 exp.) + (-0.036 *  LRRC8C-DT exp.) + (-0.113 * USP30-AS1 exp.) + (-0.064 *  AL691482.3 exp.) + (0.130 *  YTHDF3-AS1 exp.) + (0.606 *  AC000067.1 exp.) + (-0.028 *  LINC02446 exp.) + (-0.027 *  U62317.1 exp.) + (-0.012 *  MIAT exp.) + (-0.033 *  SLC12A5-AS1 exp.) + (0.038 *  LINC01235 exp.) + (1.089 *  AL138789.1 exp.). The heatmap shows the expression levels of 16 lncRNAs in BC, and we found that most of the lncRNAs were significantly highly expressed in BC, except LIPE-AS1, LINC01235, AL133467.1, LRRC8C-DT (**Figure 5D**). According to the median RS, BC patients were divided into low-risk and high-risk groups. We found that patients in the high-RS group had a poorer prognosis (**Figure 5E**). To assess the predictive ability of the model, we constructed time-dependent ROC curves, and the results showed that the CD4 TLAs-related prognostic model had high accuracy in BC patients (**Figure 5E**; AUC at 1, 3, and 5 years were 0.742, 0.751, and 0.723, respectively).

TCGA-BC patients were divided in a 1:1 ratio into a validation cohort 1 and a validation cohort 2 to further test the accuracy of the model. RS were calculated by the same algorithm, noting that high-RS was significantly associated with poorer prognosis in both validation cohort 1 (**Figure 5F**) and validation cohort 2 (**Figure 5G**). The results of AUC further confirmed the stability of the model (**Figure 5F**, AUC at 1, 3, and 5 years were 0.675, 0.646, and 0.651, respectively; **Figure 5G**, AUC at 1, 3, and 5 years were 0.701, 0.696, and 0.691, respectively). Furthermore, by univariate and multivariate COX analysis, we found that RS was an independent prognostic factor in BC patients (**Supplementary Figure 3**).

### Identification of the Role of RS in Clinical Subgroups

**Figure 6:**
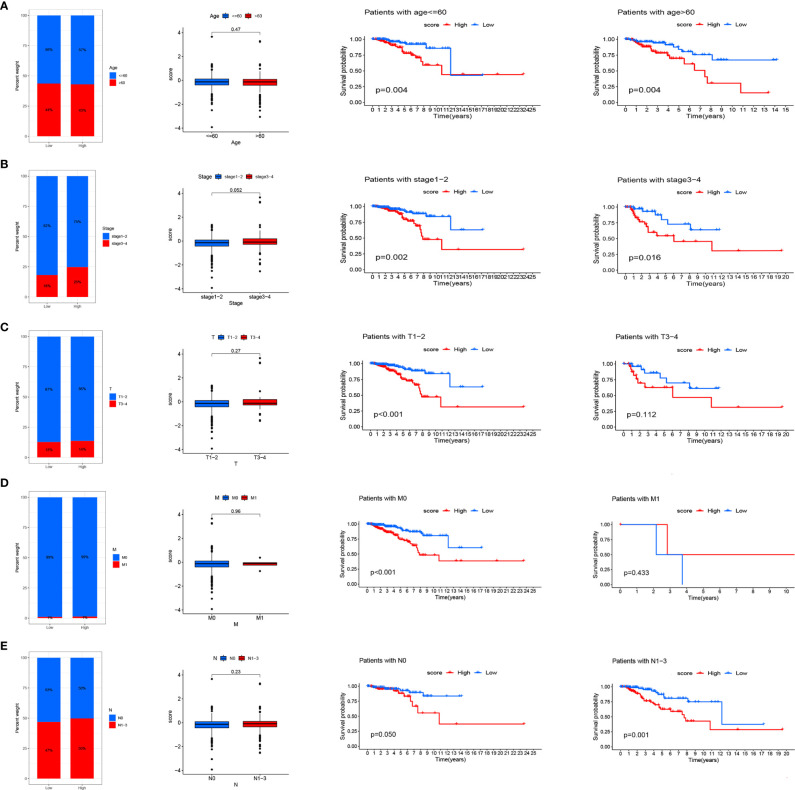
Relationship between risk scores and clinical characteristics. Relationship between risk score and Age **(A)**, Stage **(B)**, T **(C)**, M **(D)**, N **(E)**. From left to right are the distribution level exploration and survival analysis of RS in different clinical subgroups.

**Figure 7:**
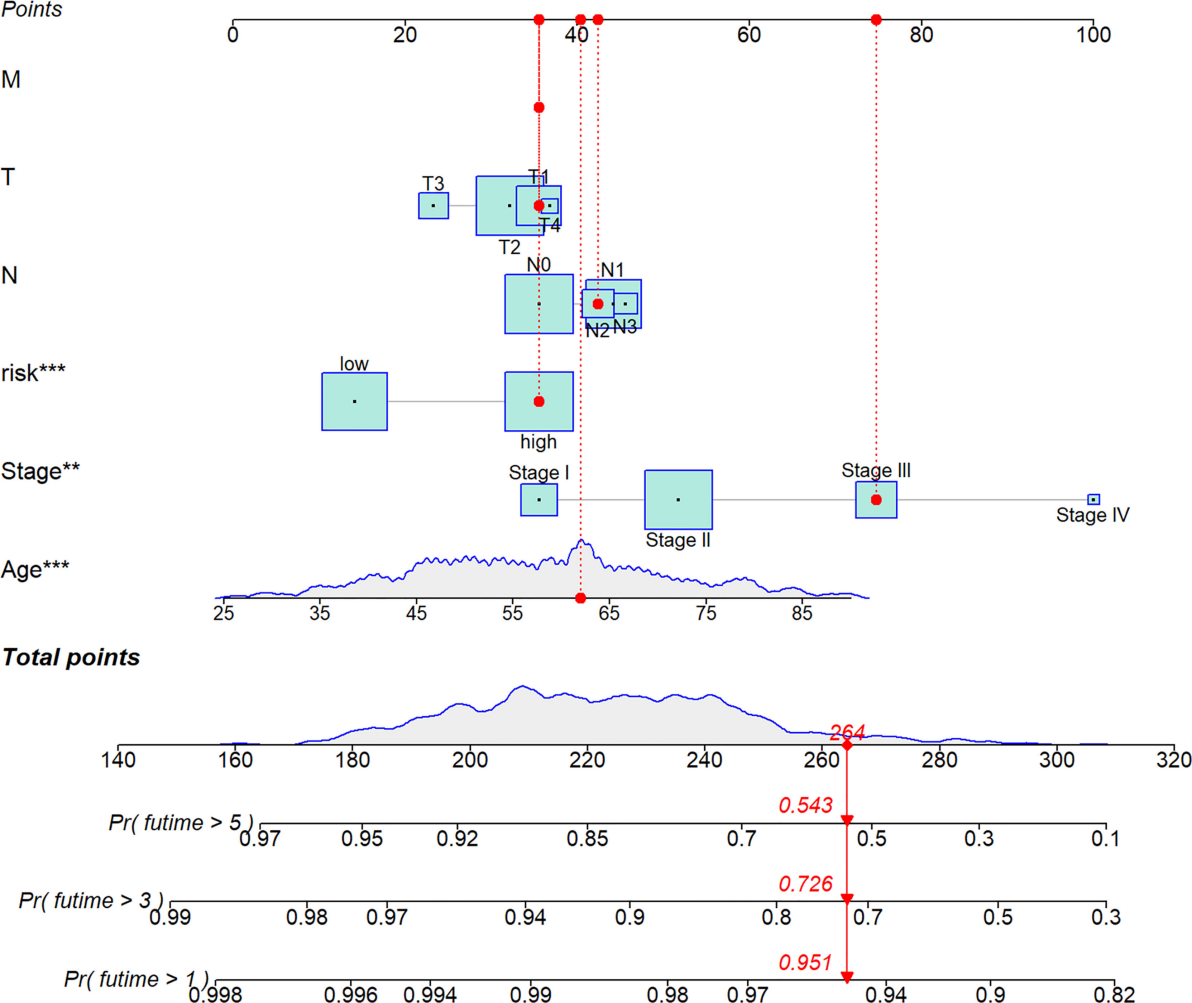
A nomogram was established integrating the RS, Age, Stage, T, M, N, for predicting 1-, 3-, and 5-year survival outcomes of BC. To calculate survival probability, identify patient values on each axis and then for each draw a vertical line upward to the points axis. Add the points for all variables and locate this sum on the total points line. * *P* < 0.05, ** *P* < 0.01, and *** *P* < 0.001.

We analyzed RS in different clinical characteristics in order to further develop the application value and test the accuracy of RS (Age, Stage, T, M, N). The results showed that there were no significant differences in RS distribution among the different clinical subgroups (**Figures 6A–E**). Furthermore, it is worth noting that RS also had a prognostic role in most clinical subgroups and that high RS was significantly associated with poorer prognosis. To improve the clinical utility of the CD4TLAs model, we constructed a predictive nomogram model incorporating clinicopathological features and RS in the TCGA dataset. The accuracy of the nomogram was checked with a calibration curve (**Supplementary Figure 4**). The results showed that RS was the main predictor (**Figure 7**).

### Analysis of the Relationship Between RS and Tumor Microenvironment

**Figure 8:**
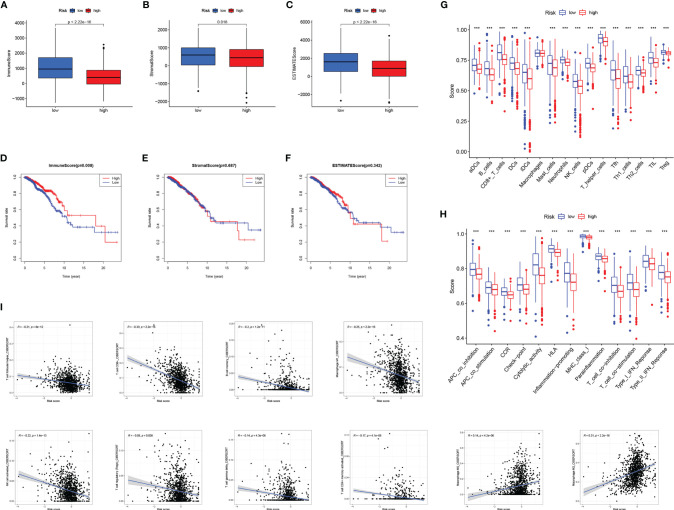
The relationship between RS and tumor microenvironment. Comparison of the immune score **(A)**, stromal score **(B)**, estimate score **(C)** in high- and low-RS groups. Relationship between immune score **(D)**, stromal score **(E)**, estimated score **(F)**, and BC prognosis. Enrichment analysis of immune cell infiltration **(G)** and immune-related pathways **(H)** in high- and low-RS groups. **(I)** Correlation analysis of immune infiltrating cells and RS. *** *P* < 0.001.

The TME has been confirmed to play a key role in the occurrence and development of BC. Exploring the relationship between RS and tumor microenvironment is helpful in the development of new functions of RS. We evaluated immune scores (**Figure 8A**), stroma scores (**Figure 8B**), and estimate scores (**Figure 8C**) in BC based on the estimate algorithm, and we found that high-RS groups tended to have lower scores. Furthermore, we explored the relationship between TME scores and prognosis, and results revealed that a high-immune score had a significant survival advantage (**Figures 8D–F**). As a further analysis, we used the ssGSEA to examine the distribution of immune cell infiltration and the enrichment of immune-related functional pathways in high- and low-RS subgroups (**Figures 8G, H**). In the low-RS group, immune infiltrating cells and immune-related pathways were significantly enriched. Another important finding was that RS was negatively correlated with most immune infiltrating cells (eg T cell follicular helper, T cell CD8^+^, B cell memory, Macrophage M1, NK cell activated, T cell regulatory, T cell gamma delta, T cell CD4^+^ memory activated), except Macrophage M0, Macrophage M2 (**Figure 8I**).

### Evaluation of the Immunotherapy Response and Chemotherapy Based on RS

**Figure 9:**
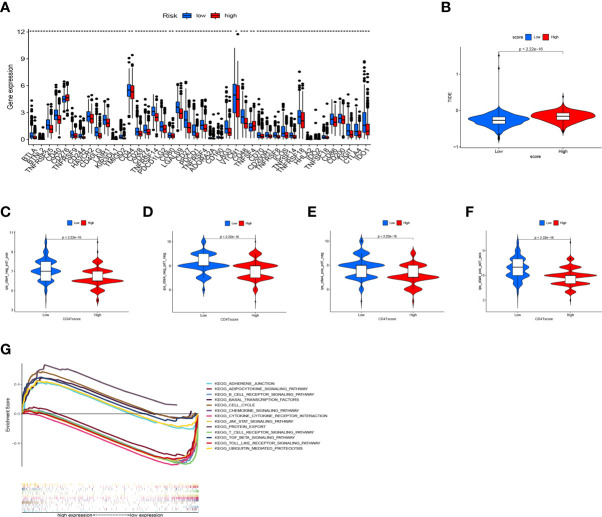
Application of RS in Immunotherapy. **(A)** Expression levels of immune checkpoints in high- and low-RS groups. **(B)** Prediction of immunotherapy response based on the TIDE algorithm. IPS score for immunotherapy. **(C)** CTLA4^−^ PD1^+^. **(D)** CTLA4^−^ PD1^−^. **(E)** CTLA4^+^ PD1^−^. **(F)** CTLA4^+^ PD1^+^. **(G)** GSEA enrichment analysis results. * *P* < 0.05, ** *P* < 0.01, and *** *P* < 0.001.

**Figure 10:**
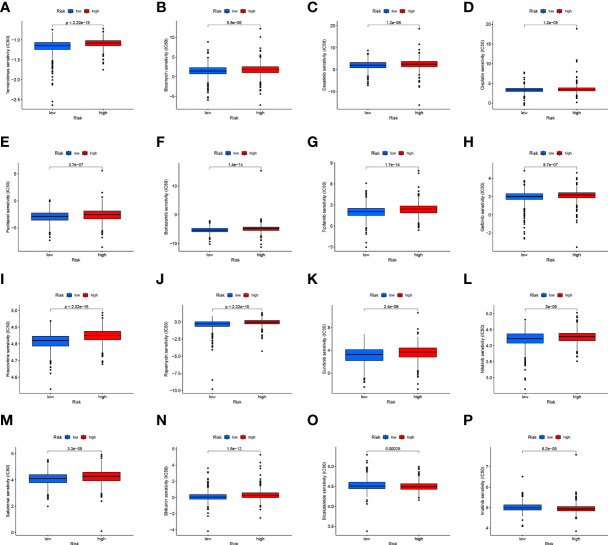
**(A–P)** Analysis of drug sensitivity in high- and low-RS groups. The distribution of IC50 score. The abscissa represents the high- and low-RS groups, and the ordinate represents the distribution of the IC50 score.

CD4TLAs-associated RS has been shown to play a very important role in the prognosis and TME of BC patients. Immunotherapy has been widely used in the clinical treatment of tumor patients, therefore, we further explored the role of RS in immunotherapy. Based on our analysis of immune checkpoint expression levels, we found that the low-RS group exhibited significantly higher expression levels of all immune checkpoints except CD276 (**Figure 9A**). In addition, we evaluated the response to immunotherapy in the high and low RS groups by the TIDE algorithm, and the results showed that the low RS group had a lower TIDE score and was more sensitive to immunotherapy (**Figure 9B**). We obtained the IPS data of BC patients through the TCIA database, and further evaluated the role of RS in IPS immunotherapy (**Figures 9C–F**). The results showed that the median line value of the low-RS group was significantly higher than that of the high-RS group, which once again proved that the low-RS group was more sensitive to immunotherapy. These findings can assist us in developing potential functions of RS as well as providing clinical assistance. GSEA enrichment analysis clarified the functional pathways that were significantly enriched in the high and low RS groups, and the results showed that they were mainly associated with cell cycle and cell receptor regulators (**Figure 9G**). We also evaluated chemotherapeutic drug responses in patients with high- and low-RS by the R package “pRRophetic”, where the half-maximal inhibitory concentration (IC50) was estimated by ridge regression and the prediction accuracy (**Figures 10A–P**). The results showed that most chemotherapeutic drugs had higher IC50 scores in the high-RS group, except for Bicalutamide (**Figure 10O**) and Imatinib (**Figure 10P**). Based on the above results, we believe that RS may help guide the clinical treatment of BC.

## Discussion

In the clinical treatment of various tumors, immunotherapy has been widely used. Advances in therapy to block inhibitory T cells checkpoint molecules such as CTLA-4 or PD-1/PD-L1 have shown clinical success, but only in a subset of BC patients (25–31). Adaptive T cell responses are very important for controlling tumor growth through the production of inflammatory cytokines and direct cytolytic targeting. CD4^+^ Tconv is also one of the major contributors to the antitumor response and can enhance patient immunity through the dendritic cell (DC) licensing and stimulation of pro-inflammatory myeloid cell programs (32–35). CD4^+^ T cells have been shown to play a crucial role in tumor progression and clinical treatment of BC patients (36–38). Therefore, studying CD4^+^ Tconv contributes to our understanding of anti-tumor and the development of novel immunotherapeutic approaches. More and more studies have shown that lncRNAs are involved in the regulation of autoimmunity and inflammation-related processes. However, research on CD4TLAs prognostic signature in patients with BC is still lacking.

As a first step in this study, we identified the major cell subsets of BC by single-cell data analysis, and we found that CD4^+^ Tconv was the largest cell subset and was significantly associated with multiple signaling pathways in BC. CD4^+^ Tconv is therefore believed to play a very important role in the development and occurrence of BC. The lncRNA network in **Figure 5A** shows the regulatory relationship between CD4^+^ Tconv-related genes and their targeted lncRNAs (CD4TLAs). LncRNAs are fundamental components of cellular processes and are involved in the occurrence and development of BC (39). CD4TLAs are thought to have important regulatory roles in BC and are associated with CD4^+^ Tconv cells. We screened and validated CD4TLAs and identified 244 CD4TLAs between tumor and normal groups. Then lncRNAs significantly associated with prognosis were screened out by univariate COX analysis, and the LASSO algorithm was used to construct a BC prognosis model and generate RS. We finally identified risk signatures for 16 lncRNAs. The accuracy and stability of the model were validated on TCGA-BRAC data, validation cohort 1, validation cohort 2, and different clinical subgroups. Having good accuracy and applicability, our model shows good performance. The results of univariate and multivariate COX analysis further validated RS as an independent prognostic factor in BC patients. The results of RS-TME relationship analysis showed that immune infiltrating cells were significantly enriched in the low-RS group, and Immune-related functional pathways are more active. We also found that RS was negatively correlated with the majority of immune cells in the TME. We believe that RS can characterize and represent TME to some extent. In addition, we further evaluated the application of RS in clinical treatment, and we found that patients in the low-RS group were more sensitive to immunotherapy and had lower IC50 scores for most chemotherapeutic drugs. This study suggest that the prognostic risk model of 16 CD4TLAs may be a reliable independent prognostic factor for breast cancer in clinical practice and can provide potential help for clinical treatment.

In recent years, precision genomic medicine has been focused on identifying specific prognostic indicators of survival based on large medical datasets with clinical outcomes. The development of prognostic risk models in BC has been increasing, and lncRNAs in BC have also attracted attention, such as ferroptosis-related lncRNAs, immune-related lncRNAs, stemness-related lncRNAs, autophagy-related lncRNA, etc (40–47). However, the role of CD4TLAs in BC prognosis remains unclear. To that end, we developed a CD4TLAs prognostic risk model to assist clinicians in determining personalized treatment strategies and to inform CD4TLAs research.

There are also some limitations to this study. First, this is a public database-based study that still needs further validation *in vivo* and *in vitro*, especially for clinical applications. Second, future studies should identify the regulatory T cells (Treg cells) apart from conventional T cells and Treg-related lncRNA signature and its relationship with prognosis and immunotherapy response in BC. This would provide a more complete picture. Finally, to further validate our bioinformatics predictions, in-depth studies of the 16 CD4TLAs, including functional experiments and molecular mechanisms, are required.

## Conclusion

In conclusion, we identified a novel BC prognostic signature consisting of 16 CD4TLAs. With further prospective validation, these signatures may become a therapeutic target for BC and inform the personalized treatment of BC patients.

## Data Availability Statement

The datasets presented in this study can be found in online repositories. The names of the repository/repositories and accession number(s) can be found in the article/**Supplementary Materials**.

## Author Contributions

SN and QH designed study, analyzed data, and wrote the manuscript. QH, JW, YP, KQ, and LL analyzed data and contributed in writing the manuscript. All authors contributed to the article and approved the submitted version.

## Funding

The work was supported by National Natural Science Foundation of China grants (82002779, 81760478); and Guangxi Provincial Natural Science Foundation of China grants (2019GXNSFAA245083); and Youth Science Foundation of Guangxi Medical University (GXMUYSF202224).

## Conflict of Interest

The authors declare that the research was conducted in the absence of any commercial or financial relationships that could be construed as a potential conflict of interest.

## Publisher’s Note

All claims expressed in this article are solely those of the authors and do not necessarily represent those of their affiliated organizations, or those of the publisher, the editors and the reviewers. Any product that may be evaluated in this article, or claim that may be made by its manufacturer, is not guaranteed or endorsed by the publisher.

## Supplementary Material

Supplementary Figure 1 Cross-shared CD4+ Tconv-related genes in the GSE110686 and GSE114727 datasets.

Supplementary Figure 2 Identification of CD4 TLAs that were significantly different between tumor and normal groups. **(A)** Volcano map. **(B)** Heatmap.

Supplementary Figure 3 RS was an independent prognostic factor in BC patients. Univariate **(A)** and Multivariate **(B)** COX analysis.

Supplementary Figure 4 The accuracy of the nomogram was checked with a calibration curve.

## Abbreviations

TCGA, The Cancer Genome Atlas; GEO, Gene-Expression Omnibus; Tconv, T conventional; CD4TLA, CD4^+^ Tconv-related lncRNA; FPKM, Fragments per Kilobase Million; TPM, Transcripts per Million; RS, risk score; TME, Tumor microenvironment; OS, Overall survival; ROC, receiver operating characteristic; TME, Tumor microenvironment; GO, Gene ontology; KEGG, Kyoto Encyclopedia of Genes and Genomes; DEGs, differentially expressed genes.
