# Supplementary figures and images for: Identification of CD4+ Conventional T Cells-Related lncRNA Signature to Improve the Prediction of Prognosis and Immunotherapy Response in Breast Cancer
Source: Front Immunol. 2022 May 4;13:880769. doi: 10.3389/fimmu.2022.880769 (PMC9114647; doi:10.3389/fimmu.2022.880769)

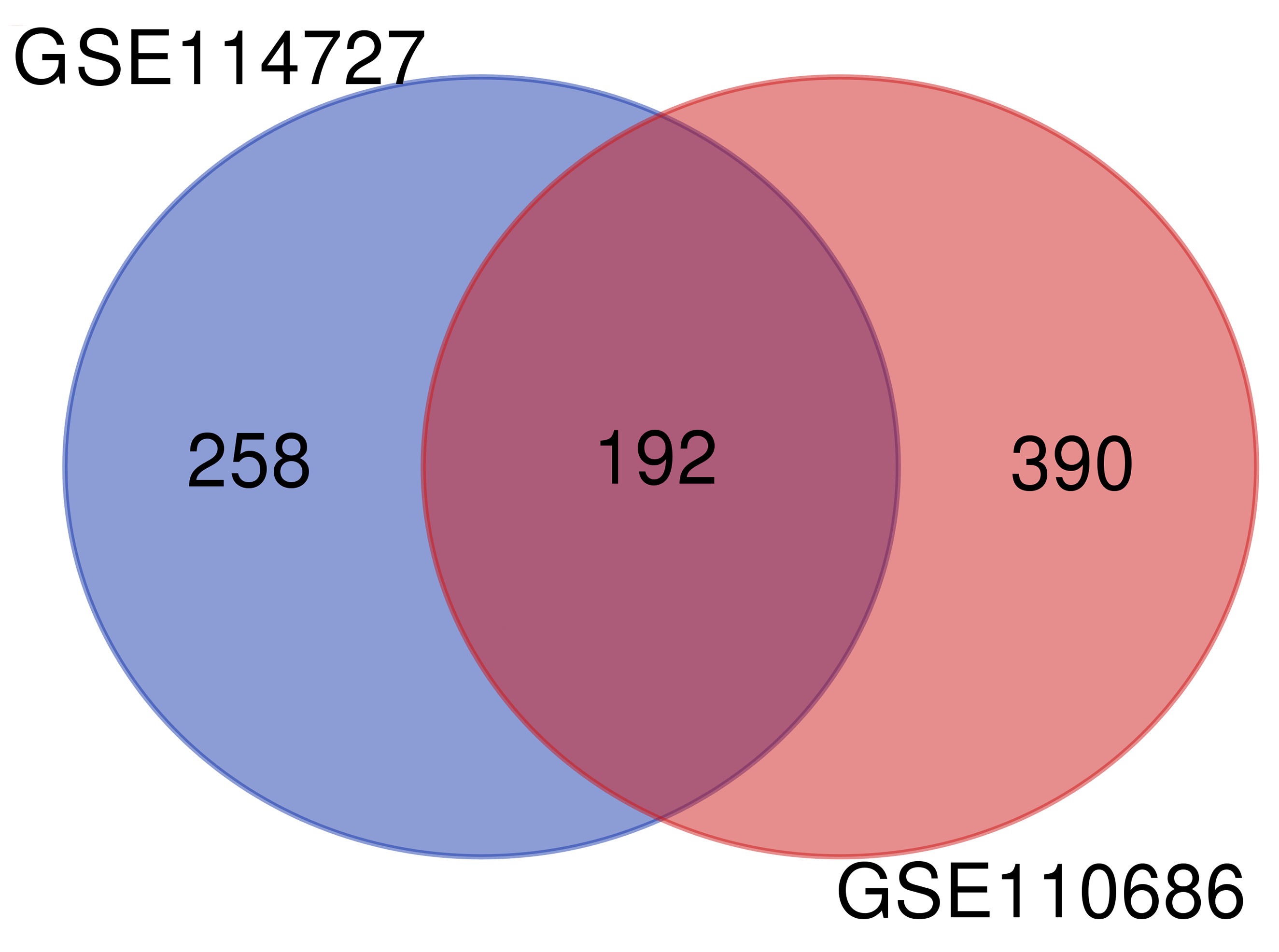

Supplement: Supplementary Figure 1 — Cross-shared CD4+ Tconv-related genes in the GSE110686 and GSE114727 datasets. [file Image_1.tif]

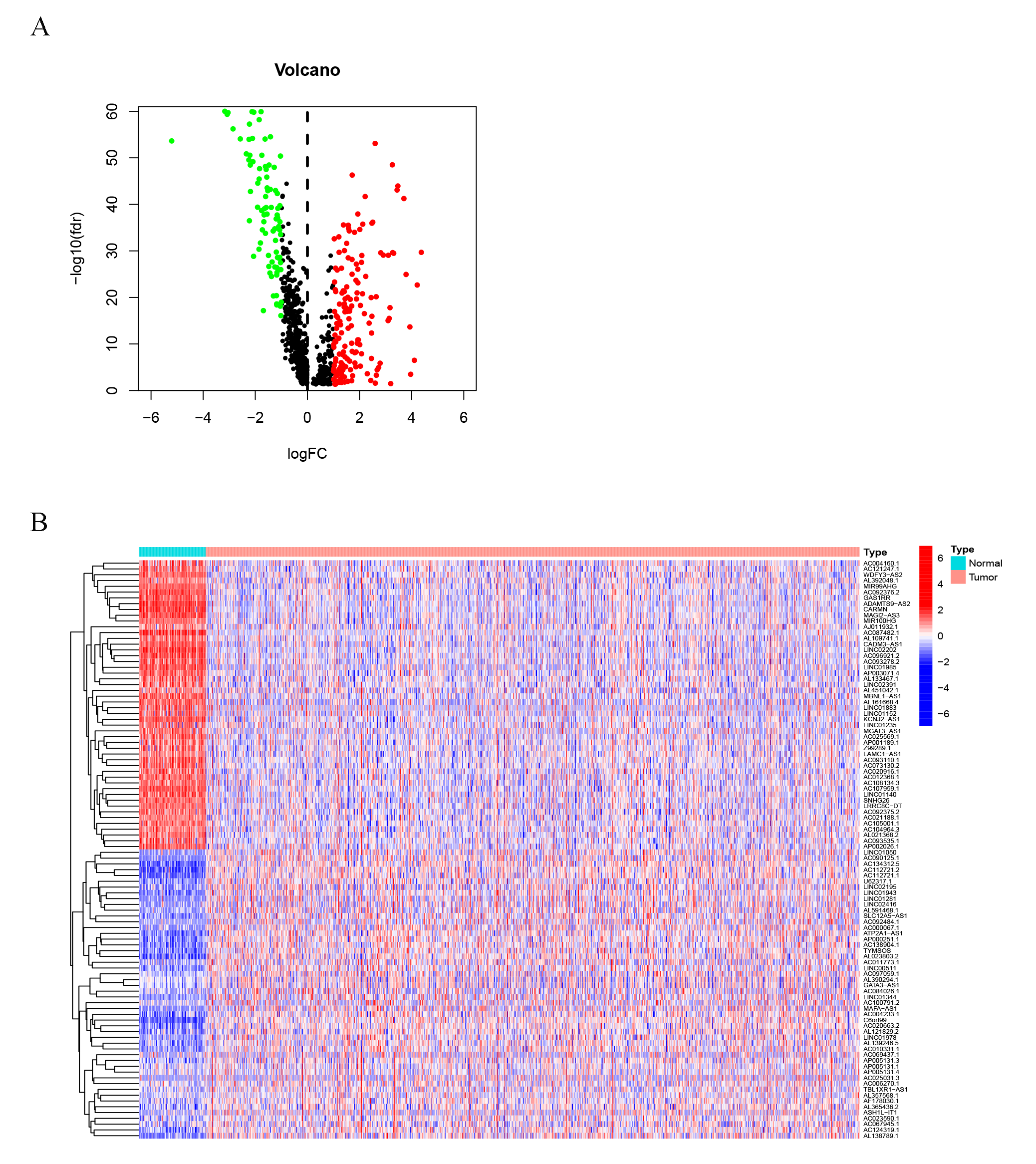

Supplement: Supplementary Figure 2 — Identification of CD4 TLAs that were significantly different between tumor and normal groups. (A) Volcano map. (B) Heatmap. [file Image_2.tif]

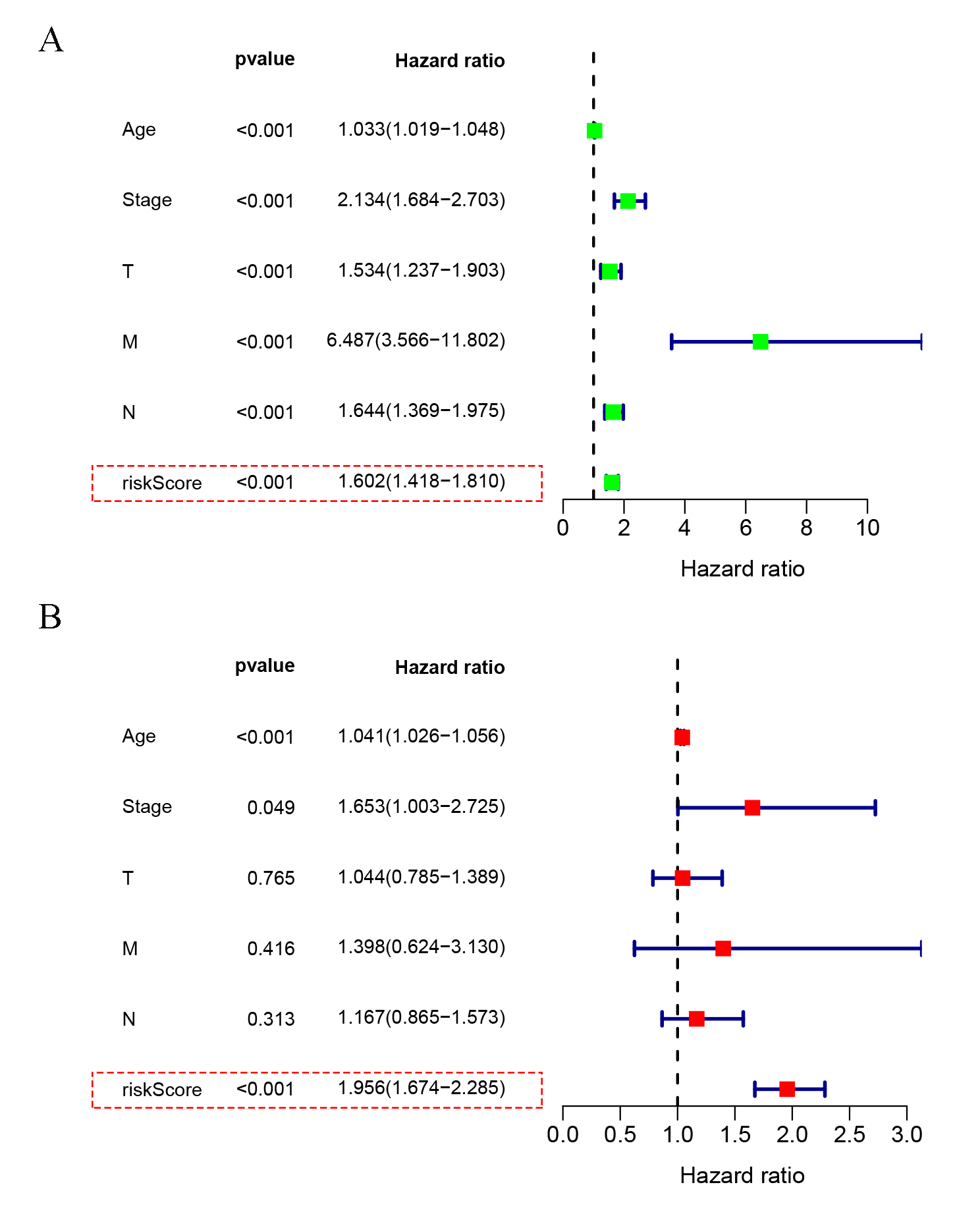

Supplement: Supplementary Figure 3 — RS was an independent prognostic factor in BC patients. Univariate (A) and Multivariate (B) COX analysis. [file Image_3.tif]

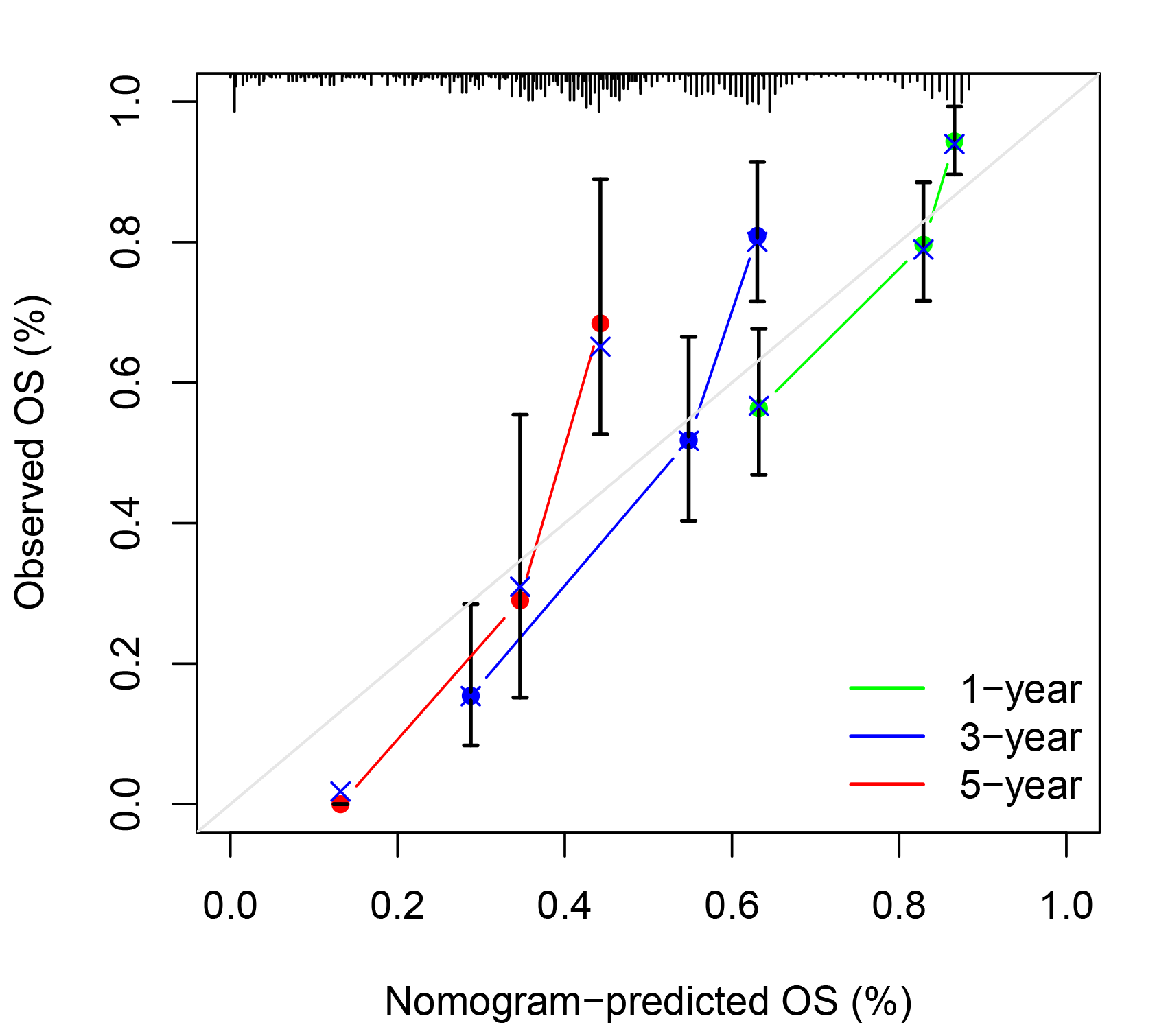

Supplement: Supplementary Figure 4 — The accuracy of the nomogram was checked with a calibration curve. [file Image_4.tif]
