# Supplementary material for: Identification of CD4+ Conventional T Cells-Related lncRNA Signature to Improve the Prediction of Prognosis and Immunotherapy Response in Breast Cancer
Source: Front Immunol. 2022 May 4;13:880769. doi: 10.3389/fimmu.2022.880769 (PMC9114647; doi:10.3389/fimmu.2022.880769)
Supplement: Supplementary file 5 [file Table_1.docx]

**Supplementary table. 1 Cross-shared CD4^+^ Tconv-related genes in the GSE110686 and GSE114727 datasets.**

| Genes |
| --- |
| MAGEH1 |
| ACTN4 |
| RGS2 |
| AC092580.4 |
| RTKN2 |
| CXCR4 |
| SLC25A3 |
| RPS18 |
| TBC1D4 |
| ADD3 |
| ANXA1 |
| GZMH |
| COTL1 |
| NOSIP |
| IRF1 |
| MIR4435-2HG |
| GATA3 |
| LSP1 |
| SH2D1A |
| CD59 |
| CXCL13 |
| CCDC50 |
| BST2 |
| PTPN22 |
| HERPUD1 |
| RPS6 |
| RUNX3 |
| GPR183 |
| FAM65B |
| EED |
| FOXP3 |
| GZMA |
| CST7 |
| TNFRSF9 |
| SOD1 |
| DUSP4 |
| RBPJ |
| CXCR3 |
| LGALS1 |
| TPI1 |
| CKLF |
| CTLA4 |
| SNX9 |
| AQP3 |
| SLC25A5 |
| ATP5G3 |
| FOS |
| IFNG |
| DBI |
| FABP5 |
| JUNB |
| CD82 |
| AC133644.2 |
| GNG5 |
| SAMSN1 |
| HLA-DPB1 |
| COX8A |
| DUSP2 |
| IDH2 |
| DYNLL1 |
| CD74 |
| C9orf16 |
| HLA-DRA |
| CTSW |
| CD63 |
| DNPH1 |
| PTMS |
| TNFRSF1B |
| GZMB |
| RASGRP2 |
| TAP1 |
| EZR |
| LAG3 |
| ARID5B |
| PMAIP1 |
| ISG20 |
| MIR155HG |
| AC017002.1 |
| MYL6 |
| GSTP1 |
| ID2 |
| HLA-DQB1 |
| GZMK |
| ITM2A |
| ZFP36L2 |
| APOBEC3C |
| SH2D2A |
| CORO1B |
| NKG7 |
| IFI27 |
| IL7R |
| ACTB |
| CALM3 |
| TNFRSF18 |
| ARPC5 |
| TNFRSF4 |
| MTHFD2 |
| TPT1 |
| CCL5 |
| LINC00152 |
| ICA1 |
| LPAR6 |
| TIGIT |
| HLA-DMA |
| LYST |
| PGAM1 |
| SLA |
| HLA-DPA1 |
| HLA-DRB1 |
| JAKMIP1 |
| PDCD1 |
| IL32 |
| HLA-B |
| TUBA1A |
| CD8B |
| PKM |
| CALR |
| LTB |
| LY6E |
| HLA-A |
| CD7 |
| CD27 |
| CFLAR |
| CMC1 |
| SIRPG |
| ETV7 |
| CD8A |
| APOBEC3G |
| RAB27A |
| DUSP1 |
| CD2 |
| MT-CO3 |
| PFN1 |
| ICOS |
| CXCR6 |
| GALM |
| CCL4 |
| TOX |
| TCF7 |
| ITM2C |
| PRF1 |
| NFKBIA |
| NDFIP2 |
| PLAC8 |
| SRGN |
| VDAC1 |
| EPSTI1 |
| HLA-C |
| GBP5 |
| CD69 |
| ITGAE |
| GAPDH |
| PHPT1 |
| CTSD |
| PSME2 |
| CCL3 |
| JUN |
| H2AFZ |
| MT2A |
| HIST1H4C |
| TIMP1 |
| ACTG1 |
| HAVCR2 |
| ZNF683 |
| ALDOA |
| MT-ND1 |
| SYTL3 |
| IL2RB |
| OASL |
| FKBP1A |
| CLIC1 |
| TXNIP |
| CD40LG |
| TYMP |
| HLA-DQA1 |
| PTTG1 |
| KLRD1 |
| ANXA5 |
| BTG3 |
| BATF |
| CARD16 |
| STMN1 |
| RAN |
| EEF1A1 |
| CCR7 |
| RGS1 |
| KLRB1 |
| LINC00861 |
| UCP2 |
| BIRC3 |
| GNLY |
| CTSC |
