# Supplementary material for: Identification of CD4+ Conventional T Cells-Related lncRNA Signature to Improve the Prediction of Prognosis and Immunotherapy Response in Breast Cancer
Source: Front Immunol. 2022 May 4;13:880769. doi: 10.3389/fimmu.2022.880769 (PMC9114647; doi:10.3389/fimmu.2022.880769)
Supplement: Supplementary file 6 [file Table_2.docx]

**Supplementary table. 2 Identification of CD4TLAs.**

| gene | conMean | treatMean | logFC | pValue | fdr |
| --- | --- | --- | --- | --- | --- |
| LINC01063 | 0.227113 | 0.603969 | 1.411066 | 3.36E-19 | 1.15E-18 |
| AC245014.3 | 0.294701 | 0.648967 | 1.138893 | 0.000386 | 0.000464 |
| AC084026.1 | 0.057783 | 0.384327 | 2.733628 | 8.11E-06 | 1.14E-05 |
| MMP2-AS1 | 0.148989 | 0.449919 | 1.594462 | 1.33E-16 | 3.76E-16 |
| AC027449.1 | 0.245721 | 0.096081 | -1.35471 | 3.45E-29 | 2.41E-28 |
| AC020765.2 | 0.431618 | 0.872249 | 1.014986 | 2.54E-10 | 4.97E-10 |
| AL136115.2 | 0.178884 | 0.461625 | 1.367695 | 2.29E-06 | 3.35E-06 |
| AC097059.1 | 0.169829 | 1.451691 | 3.095576 | 3.44E-16 | 9.53E-16 |
| AC007255.1 | 0.400994 | 1.303194 | 1.7004 | 1.97E-19 | 6.87E-19 |
| AL683807.1 | 0.161337 | 0.504212 | 1.643949 | 6.46E-21 | 2.53E-20 |
| AC105001.1 | 0.432923 | 0.144584 | -1.5822 | 1.05E-49 | 3.04E-48 |
| AC004233.1 | 0.065796 | 0.279828 | 2.088478 | 1.26E-30 | 9.65E-30 |
| AC011445.1 | 0.141343 | 0.341032 | 1.270708 | 2.93E-15 | 7.58E-15 |
| DLEU2 | 0.397955 | 0.924418 | 1.21594 | 2.41E-31 | 2.04E-30 |
| CYTOR | 2.231904 | 4.716762 | 1.079522 | 7.99E-28 | 5.15E-27 |
| AC108463.3 | 0.082285 | 0.164919 | 1.003046 | 5.13E-05 | 6.78E-05 |
| AC022706.1 | 2.087793 | 0.950103 | -1.13582 | 4.57E-41 | 7.21E-40 |
| MAGI2-AS3 | 3.977698 | 0.911518 | -2.12559 | 4.39E-63 | 1.23E-60 |
| UBE2R2-AS1 | 0.085204 | 0.218692 | 1.359911 | 0.000818 | 0.000958 |
| AC112721.2 | 0.063894 | 0.693805 | 3.440781 | 3.75E-45 | 8.26E-44 |
| AC087482.1 | 1.803385 | 0.048811 | -5.20736 | 4.22E-56 | 2.36E-54 |
| AC008764.2 | 7.563109 | 3.617674 | -1.06392 | 2.80E-36 | 3.04E-35 |
| AC010531.3 | 0.221166 | 0.097969 | -1.17473 | 1.15E-19 | 4.07E-19 |
| AC096921.2 | 1.539218 | 0.329508 | -2.22381 | 5.40E-60 | 5.65E-58 |
| AC004884.2 | 0.104275 | 0.377841 | 1.857391 | 5.67E-06 | 8.07E-06 |
| AC022893.3 | 0.228686 | 0.114249 | -1.00119 | 2.83E-20 | 1.07E-19 |
| AC020916.1 | 21.2119 | 6.661779 | -1.67089 | 4.08E-38 | 5.42E-37 |
| AC134312.5 | 0.08814 | 0.844132 | 3.259592 | 8.79E-51 | 3.20E-49 |
| AC109322.1 | 0.649631 | 1.685088 | 1.375131 | 8.18E-23 | 3.83E-22 |
| AC012368.1 | 1.21842 | 0.34158 | -1.83471 | 7.24E-50 | 2.16E-48 |
| STAM-AS1 | 0.07656 | 0.157843 | 1.043818 | 3.42E-18 | 1.08E-17 |
| AC068580.3 | 0.408722 | 1.340308 | 1.713373 | 1.77E-26 | 1.03E-25 |
| AC008115.3 | 0.945712 | 2.182982 | 1.206828 | 2.65E-12 | 5.88E-12 |
| AC137630.3 | 0.168082 | 0.357216 | 1.087633 | 9.71E-08 | 1.58E-07 |
| AC037198.1 | 1.073516 | 4.044334 | 1.913558 | 3.40E-11 | 7.02E-11 |
| AC104964.3 | 0.740924 | 0.236255 | -1.64898 | 5.71E-41 | 8.84E-40 |
| WDFY3-AS2 | 1.098612 | 0.411127 | -1.41802 | 3.64E-57 | 3.05E-55 |
| LINC01359 | 0.337852 | 0.162614 | -1.05494 | 1.11E-41 | 1.94E-40 |
| AL021368.2 | 0.665598 | 0.247979 | -1.42443 | 2.72E-45 | 6.16E-44 |
| AL109741.1 | 1.68461 | 0.450041 | -1.90429 | 1.10E-46 | 2.79E-45 |
| AC073130.2 | 0.680864 | 0.188624 | -1.85185 | 1.38E-47 | 3.60E-46 |
| AL049838.1 | 1.794519 | 0.814692 | -1.13927 | 6.08E-39 | 8.48E-38 |
| LINC01140 | 1.231938 | 0.269506 | -2.19254 | 1.05E-50 | 3.53E-49 |
| LINC01857 | 0.389915 | 1.110118 | 1.50948 | 1.07E-23 | 5.37E-23 |
| SOX9-AS1 | 1.44214 | 0.554813 | -1.37814 | 5.51E-26 | 3.12E-25 |
| AC022211.1 | 0.160968 | 0.398389 | 1.307405 | 1.65E-13 | 3.99E-13 |
| AC023590.1 | 0.041437 | 0.160704 | 1.955423 | 6.27E-12 | 1.34E-11 |
| UBE2Q1-AS1 | 0.173932 | 0.380196 | 1.128222 | 1.54E-15 | 4.00E-15 |
| AC007728.3 | 0.060608 | 0.164336 | 1.439064 | 1.60E-10 | 3.16E-10 |
| AC040904.1 | 0.045364 | 0.142678 | 1.653139 | 2.56E-05 | 3.46E-05 |
| LINC01281 | 0.012189 | 0.117731 | 3.271887 | 2.91E-31 | 2.39E-30 |
| AC040169.1 | 0.906113 | 1.849749 | 1.029566 | 2.95E-06 | 4.29E-06 |
| AP005131.4 | 0.032913 | 0.301836 | 3.197035 | 0.032849 | 0.033327 |
| AL023803.2 | 0.030886 | 0.342557 | 3.471309 | 4.80E-46 | 1.15E-44 |
| MIR155HG | 0.438924 | 0.933428 | 1.088569 | 1.63E-11 | 3.42E-11 |
| AC006270.1 | 0.064623 | 0.404048 | 2.644398 | 0.000439 | 0.000526 |
| AL121832.2 | 1.132308 | 2.688556 | 1.247564 | 5.22E-16 | 1.41E-15 |
| AL161668.4 | 0.58071 | 0.122343 | -2.24689 | 7.16E-52 | 2.85E-50 |
| CARMN | 2.053164 | 0.284048 | -2.85364 | 6.57E-59 | 6.11E-57 |
| AC087239.1 | 0.153403 | 0.473064 | 1.624709 | 1.09E-18 | 3.61E-18 |
| AC106795.5 | 0.036585 | 0.128874 | 1.816616 | 4.19E-09 | 7.60E-09 |
| SENCR | 1.089598 | 0.438275 | -1.31389 | 4.98E-36 | 5.28E-35 |
| AL157838.1 | 0.311506 | 0.863268 | 1.470547 | 5.35E-19 | 1.80E-18 |
| LINC01615 | 0.2017 | 0.770956 | 1.934438 | 1.38E-27 | 8.64E-27 |
| NRIR | 0.186274 | 0.37423 | 1.0065 | 0.003724 | 0.004167 |
| LINC01978 | 0.030978 | 0.134098 | 2.113969 | 3.72E-22 | 1.63E-21 |
| LINC02345 | 0.06682 | 0.156344 | 1.226368 | 8.33E-09 | 1.48E-08 |
| AC109361.2 | 0.306918 | 0.714218 | 1.21851 | 2.02E-05 | 2.75E-05 |
| AL807757.2 | 0.072502 | 0.1622 | 1.161676 | 0.010548 | 0.011233 |
| AP005131.1 | 0.054777 | 0.333362 | 2.605439 | 0.025793 | 0.026392 |
| AC007750.1 | 0.053618 | 0.143244 | 1.41768 | 6.78E-20 | 2.49E-19 |
| SNHG25 | 0.639166 | 2.325278 | 1.86314 | 3.95E-25 | 2.12E-24 |
| AC092484.1 | 0.016267 | 0.248398 | 3.932642 | 8.61E-15 | 2.17E-14 |
| AC093535.1 | 0.861084 | 0.237662 | -1.85724 | 4.85E-32 | 4.37E-31 |
| SIDT1-AS1 | 0.12314 | 0.286063 | 1.216037 | 0.015632 | 0.016294 |
| AP003419.3 | 0.404325 | 1.193451 | 1.561551 | 1.65E-14 | 4.13E-14 |
| MIR210HG | 0.551071 | 1.612047 | 1.548584 | 2.25E-21 | 9.23E-21 |
| AL133467.1 | 1.22741 | 0.262451 | -2.2255 | 2.48E-38 | 3.34E-37 |
| LINC01943 | 0.129402 | 0.494724 | 1.934766 | 7.96E-40 | 1.19E-38 |
| LIPE-AS1 | 1.100051 | 0.47087 | -1.22417 | 5.92E-34 | 5.70E-33 |
| DDX11-AS1 | 0.15933 | 0.426356 | 1.420036 | 9.86E-32 | 8.78E-31 |
| AL392048.1 | 0.303595 | 0.103822 | -1.54803 | 8.42E-40 | 1.24E-38 |
| LINC00092 | 0.579157 | 0.221534 | -1.38643 | 2.73E-41 | 4.57E-40 |
| AC092376.2 | 0.665374 | 0.159988 | -2.05621 | 7.57E-63 | 1.58E-60 |
| LINC01093 | 0.241077 | 0.106421 | -1.17971 | 6.87E-20 | 2.51E-19 |
| AC015819.1 | 0.385116 | 0.902873 | 1.22923 | 7.45E-20 | 2.70E-19 |
| AC025031.3 | 0.026963 | 0.148009 | 2.456647 | 7.90E-08 | 1.30E-07 |
| ADAMTS9-AS2 | 0.780376 | 0.092364 | -3.07877 | 3.21E-62 | 4.47E-60 |
| EIF1B-AS1 | 0.360753 | 0.176168 | -1.03406 | 9.97E-53 | 4.17E-51 |
| AL139246.5 | 0.439577 | 1.68467 | 1.938277 | 1.40E-24 | 7.31E-24 |
| AC000403.1 | 0.547566 | 0.245819 | -1.15544 | 2.35E-44 | 4.69E-43 |
| AC012213.3 | 0.128509 | 0.318233 | 1.308214 | 8.35E-28 | 5.29E-27 |
| AC110792.3 | 0.896792 | 0.438733 | -1.03143 | 4.91E-29 | 3.34E-28 |
| AC090125.1 | 0.013571 | 0.280665 | 4.370286 | 2.47E-31 | 2.05E-30 |
| AC005757.1 | 0.038796 | 0.113664 | 1.550781 | 0.010113 | 0.010783 |
| LINC00504 | 1.482586 | 2.983504 | 1.008892 | 1.70E-05 | 2.35E-05 |
| LINC01985 | 0.787543 | 0.087559 | -3.16904 | 1.21E-63 | 1.01E-60 |
| MGAT3-AS1 | 0.312272 | 0.082942 | -1.91262 | 2.41E-41 | 4.12E-40 |
| AC092375.2 | 0.3193 | 0.09455 | -1.75576 | 1.42E-40 | 2.16E-39 |
| NARF-AS1 | 0.042527 | 0.130488 | 1.617455 | 2.74E-18 | 8.84E-18 |
| AP001189.1 | 0.874511 | 0.25075 | -1.80223 | 2.07E-33 | 1.95E-32 |
| BRWD1-AS1 | 0.09427 | 0.231119 | 1.293768 | 7.17E-07 | 1.08E-06 |
| AC124312.5 | 2.548449 | 1.071879 | -1.24948 | 4.37E-28 | 2.91E-27 |
| IDH2-DT | 0.176997 | 0.430295 | 1.281601 | 5.60E-06 | 7.99E-06 |
| AC024075.1 | 4.610413 | 2.167153 | -1.0891 | 5.22E-30 | 3.77E-29 |
| AC121247.1 | 0.590241 | 0.186473 | -1.66234 | 1.26E-39 | 1.82E-38 |
| LRRC8C-DT | 0.775583 | 0.279801 | -1.47088 | 1.01E-50 | 3.52E-49 |
| AP003392.3 | 0.057892 | 0.167248 | 1.530543 | 3.10E-18 | 9.86E-18 |
| CADM3-AS1 | 0.840075 | 0.14099 | -2.57493 | 1.25E-56 | 8.69E-55 |
| C1orf220 | 0.205686 | 0.477587 | 1.21532 | 1.18E-15 | 3.10E-15 |
| AC004160.1 | 0.370043 | 0.114768 | -1.68897 | 2.09E-18 | 6.87E-18 |
| POLH-AS1 | 0.300456 | 0.783403 | 1.3826 | 2.21E-37 | 2.68E-36 |
| MIR4435-2HG | 1.340555 | 2.737267 | 1.029906 | 2.58E-34 | 2.54E-33 |
| C6orf99 | 0.258286 | 1.564875 | 2.599005 | 1.56E-55 | 8.14E-54 |
| LINC02202 | 1.515441 | 0.183592 | -3.04516 | 1.16E-62 | 1.93E-60 |
| AC004223.2 | 0.053736 | 0.130022 | 1.27478 | 0.002814 | 0.003166 |
| AC103769.1 | 0.076675 | 0.24895 | 1.699032 | 0.007391 | 0.007972 |
| AL390719.2 | 1.325653 | 2.664403 | 1.007109 | 6.22E-11 | 1.26E-10 |
| AC022079.1 | 0.074135 | 0.153692 | 1.051818 | 0.041129 | 0.041376 |
| ASH1L-IT1 | 0.019855 | 0.137277 | 2.789536 | 9.12E-07 | 1.36E-06 |
| AC025569.1 | 0.278054 | 0.091525 | -1.60313 | 1.12E-43 | 2.10E-42 |
| TXNDC12-AS1 | 0.047558 | 0.117718 | 1.307586 | 0.017468 | 0.018163 |
| AC107959.1 | 0.802291 | 0.233852 | -1.77853 | 4.41E-63 | 1.23E-60 |
| AC093424.1 | 0.243326 | 0.10836 | -1.16705 | 7.09E-27 | 4.27E-26 |
| U62317.2 | 2.528661 | 5.20318 | 1.04102 | 9.23E-25 | 4.89E-24 |
| LINC02195 | 0.057577 | 0.32019 | 2.475356 | 3.83E-17 | 1.13E-16 |
| LINC01711 | 0.342665 | 0.93409 | 1.446764 | 5.24E-21 | 2.07E-20 |
| GATA3-AS1 | 0.925966 | 5.103085 | 2.462338 | 1.89E-13 | 4.50E-13 |
| AC010331.1 | 0.109794 | 0.462828 | 2.07567 | 4.24E-29 | 2.91E-28 |
| GAS1RR | 0.98774 | 0.209198 | -2.23926 | 1.79E-56 | 1.07E-54 |
| AL365436.2 | 0.097926 | 0.445241 | 2.184827 | 9.98E-18 | 3.05E-17 |
| AL591468.1 | 0.024451 | 0.219974 | 3.169357 | 4.73E-19 | 1.60E-18 |
| AL450344.3 | 0.208009 | 0.093893 | -1.14755 | 3.58E-30 | 2.63E-29 |
| AC011447.3 | 0.145086 | 0.302979 | 1.062311 | 1.17E-11 | 2.46E-11 |
| AC091057.1 | 0.215933 | 0.643885 | 1.576218 | 1.22E-36 | 1.39E-35 |
| AC138696.2 | 0.74875 | 2.453342 | 1.712193 | 9.79E-30 | 7.00E-29 |
| C5orf66 | 0.112989 | 0.236753 | 1.067204 | 4.62E-23 | 2.21E-22 |
| LINC01883 | 0.545865 | 0.120066 | -2.18471 | 8.46E-45 | 1.73E-43 |
| AP002026.1 | 0.606326 | 0.180489 | -1.74818 | 6.38E-53 | 2.81E-51 |
| LINC01094 | 0.663341 | 1.464613 | 1.142698 | 2.12E-27 | 1.30E-26 |
| AL359220.1 | 0.37397 | 0.174172 | -1.10241 | 5.24E-37 | 6.11E-36 |
| AJ011932.1 | 0.38678 | 0.142698 | -1.43855 | 9.57E-27 | 5.68E-26 |
| LINC02391 | 0.721871 | 0.217983 | -1.72753 | 2.76E-36 | 3.04E-35 |
| AC067945.1 | 0.040849 | 0.167058 | 2.031969 | 4.10E-06 | 5.91E-06 |
| MIR100HG | 5.427249 | 1.760032 | -1.62462 | 1.44E-56 | 9.27E-55 |
| USP30-AS1 | 0.427467 | 1.089201 | 1.349384 | 1.07E-08 | 1.87E-08 |
| AC127024.3 | 0.059797 | 0.198972 | 1.734427 | 0.000575 | 0.000683 |
| AC011773.1 | 0.033419 | 0.134517 | 2.009039 | 7.05E-11 | 1.42E-10 |
| AC025175.1 | 0.995437 | 0.488272 | -1.02765 | 2.94E-35 | 3.00E-34 |
| LINC02516 | 0.055312 | 0.147747 | 1.417465 | 0.001088 | 0.001257 |
| MAL2-AS1 | 0.059386 | 0.226752 | 1.932912 | 6.23E-12 | 1.33E-11 |
| TYMSOS | 0.31217 | 1.361044 | 2.12431 | 1.48E-37 | 1.82E-36 |
| AL359878.2 | 0.105862 | 0.226612 | 1.098037 | 0.000134 | 0.000171 |
| AF178030.1 | 0.117657 | 2.024602 | 4.104983 | 2.05E-07 | 3.25E-07 |
| AC015813.4 | 0.15393 | 0.562971 | 1.870783 | 2.88E-09 | 5.32E-09 |
| LINC02273 | 0.117478 | 0.247862 | 1.077144 | 0.043109 | 0.043212 |
| AC098869.2 | 0.156228 | 0.329837 | 1.078102 | 3.58E-07 | 5.54E-07 |
| AL662844.4 | 1.412902 | 0.605288 | -1.22297 | 4.94E-45 | 1.06E-43 |
| AP001453.2 | 0.608618 | 2.130052 | 1.807279 | 9.79E-36 | 1.02E-34 |
| AC021188.1 | 0.671315 | 0.227977 | -1.5581 | 5.20E-48 | 1.40E-46 |
| Z99289.1 | 0.515832 | 0.168985 | -1.61 | 1.64E-35 | 1.69E-34 |
| AC093110.1 | 2.685384 | 0.529094 | -2.34353 | 2.87E-53 | 1.41E-51 |
| AL691482.3 | 0.681565 | 1.466864 | 1.105813 | 9.47E-05 | 0.000122 |
| AC004687.1 | 0.387198 | 0.825742 | 1.092621 | 0.000695 | 0.00082 |
| MBNL1-AS1 | 1.590197 | 0.520825 | -1.61033 | 2.26E-50 | 7.28E-49 |
| AP005131.3 | 0.088434 | 0.778749 | 3.13848 | 1.13E-16 | 3.22E-16 |
| AC124319.1 | 0.166533 | 0.785506 | 2.23781 | 5.40E-26 | 3.08E-25 |
| AC083880.1 | 0.34481 | 0.918689 | 1.413777 | 1.23E-22 | 5.58E-22 |
| SNHG26 | 1.638649 | 0.385037 | -2.08944 | 1.80E-51 | 6.85E-50 |
| AL021026.1 | 0.302166 | 0.126871 | -1.25198 | 1.61E-36 | 1.82E-35 |
| YTHDF3-AS1 | 0.901905 | 1.879175 | 1.059052 | 5.33E-13 | 1.23E-12 |
| AC048341.1 | 0.79849 | 0.382773 | -1.06078 | 3.30E-30 | 2.46E-29 |
| AC093423.2 | 0.450626 | 0.201687 | -1.15981 | 1.37E-39 | 1.94E-38 |
| AC104462.1 | 0.055552 | 0.180067 | 1.696628 | 3.40E-11 | 7.02E-11 |
| AC011503.2 | 0.369258 | 0.927311 | 1.328426 | 2.60E-22 | 1.15E-21 |
| AC100791.2 | 0.030034 | 0.147641 | 2.297426 | 0.000201 | 0.00025 |
| AC011465.1 | 0.137106 | 0.396247 | 1.53111 | 1.01E-07 | 1.64E-07 |
| AC020663.2 | 0.219453 | 0.885031 | 2.011818 | 2.25E-36 | 2.51E-35 |
| LINC01703 | 0.654157 | 1.42017 | 1.118354 | 1.67E-14 | 4.16E-14 |
| MAFA-AS1 | 0.019259 | 0.357392 | 4.213879 | 4.20E-24 | 2.14E-23 |
| AP003071.4 | 0.924651 | 0.200304 | -2.20671 | 6.01E-53 | 2.79E-51 |
| AL451042.1 | 0.247013 | 0.088542 | -1.48014 | 3.35E-28 | 2.24E-27 |
| AC004816.2 | 0.457129 | 0.202665 | -1.17351 | 5.86E-28 | 3.83E-27 |
| AP000251.1 | 0.1648 | 0.917014 | 2.476227 | 8.82E-38 | 1.12E-36 |
| FO680682.1 | 0.04777 | 0.147008 | 1.621728 | 3.13E-07 | 4.89E-07 |
| LINC02416 | 0.028407 | 0.146601 | 2.367553 | 1.29E-15 | 3.37E-15 |
| AC010503.4 | 7.941588 | 18.33085 | 1.206774 | 9.67E-35 | 9.64E-34 |
| AL163051.1 | 0.262922 | 0.558014 | 1.085667 | 1.19E-22 | 5.44E-22 |
| LINC02574 | 0.045675 | 0.150348 | 1.71883 | 2.11E-09 | 3.92E-09 |
| LINC00511 | 0.11204 | 0.788597 | 2.815276 | 3.16E-31 | 2.57E-30 |
| AC138904.1 | 0.109989 | 0.625453 | 2.507545 | 5.31E-38 | 6.84E-37 |
| AC027228.2 | 0.137217 | 0.379763 | 1.468642 | 5.51E-06 | 7.89E-06 |
| LINC01050 | 0.024728 | 0.154079 | 2.639484 | 1.73E-21 | 7.12E-21 |
| KCNJ2-AS1 | 0.803678 | 0.186446 | -2.10786 | 8.94E-57 | 6.80E-55 |
| AL078587.1 | 0.066303 | 0.180449 | 1.444453 | 3.53E-08 | 5.97E-08 |
| LINC01055 | 0.21017 | 0.102209 | -1.04003 | 2.16E-19 | 7.49E-19 |
| TBL1XR1-AS1 | 0.0322 | 0.20585 | 2.676458 | 2.68E-05 | 3.61E-05 |
| AC108134.4 | 0.759198 | 2.060591 | 1.440511 | 4.07E-18 | 1.26E-17 |
| LINC01152 | 3.016491 | 0.720531 | -2.06574 | 1.95E-30 | 1.47E-29 |
| AC000067.1 | 0.028304 | 0.154282 | 2.446472 | 2.63E-21 | 1.07E-20 |
| MIR200CHG | 5.086171 | 11.37712 | 1.161484 | 6.95E-17 | 2.01E-16 |
| LINC02446 | 0.345059 | 0.927347 | 1.426267 | 0.014675 | 0.015334 |
| AC004585.1 | 0.233368 | 0.857195 | 1.877017 | 1.02E-28 | 6.91E-28 |
| HCG11 | 8.282762 | 3.427974 | -1.27276 | 3.51E-50 | 1.09E-48 |
| MIR99AHG | 2.217613 | 0.614935 | -1.8505 | 5.45E-61 | 6.51E-59 |
| LINC01344 | 0.01483 | 0.204452 | 3.785193 | 2.01E-26 | 1.16E-25 |
| AL031846.2 | 0.653136 | 0.322179 | -1.01952 | 1.70E-27 | 1.06E-26 |
| LINC02100 | 0.129692 | 0.332761 | 1.359404 | 2.46E-05 | 3.33E-05 |
| TMPO-AS1 | 0.303504 | 0.99892 | 1.718651 | 1.84E-48 | 5.14E-47 |
| AC112721.1 | 0.028519 | 0.371381 | 3.702909 | 3.22E-43 | 5.73E-42 |
| LINC02550 | 0.556814 | 0.244524 | -1.18722 | 9.69E-22 | 4.10E-21 |
| AC093799.1 | 1.112252 | 0.484695 | -1.19833 | 9.58E-39 | 1.31E-37 |
| U62317.1 | 0.489278 | 3.680466 | 2.911161 | 1.04E-30 | 8.18E-30 |
| AL445490.1 | 0.147586 | 0.363858 | 1.301823 | 0.000155 | 0.000197 |
| MIAT | 0.26807 | 0.750771 | 1.485764 | 5.40E-19 | 1.82E-18 |
| AC012073.1 | 0.374084 | 1.108759 | 1.567512 | 2.57E-37 | 3.08E-36 |
| AC130651.1 | 0.065211 | 0.151608 | 1.217155 | 5.55E-05 | 7.31E-05 |
| AC068580.1 | 0.364945 | 1.36771 | 1.906012 | 2.36E-22 | 1.05E-21 |
| AL390294.1 | 0.440569 | 1.859243 | 2.077275 | 7.20E-09 | 1.28E-08 |
| SLC12A5-AS1 | 0.021597 | 0.185901 | 3.105667 | 1.13E-30 | 8.86E-30 |
| AC007336.2 | 0.055457 | 0.178251 | 1.684473 | 4.82E-15 | 1.23E-14 |
| AL133243.1 | 0.047319 | 0.121238 | 1.357338 | 2.68E-05 | 3.61E-05 |
| HLA-F-AS1 | 0.78696 | 0.379032 | -1.05397 | 4.29E-38 | 5.61E-37 |
| VCAN-AS1 | 0.045361 | 0.160435 | 1.822455 | 8.26E-07 | 1.24E-06 |
| AP005205.2 | 0.30972 | 0.152902 | -1.01836 | 2.69E-17 | 8.05E-17 |
| AC093278.2 | 4.472317 | 1.53389 | -1.54383 | 1.25E-45 | 2.90E-44 |
| AL360270.2 | 0.071236 | 0.15393 | 1.111602 | 3.19E-17 | 9.46E-17 |
| LAMC1-AS1 | 1.123983 | 0.375096 | -1.58329 | 2.95E-41 | 4.84E-40 |
| LINC01235 | 5.626359 | 1.997096 | -1.4943 | 1.22E-30 | 9.44E-30 |
| AL121829.2 | 0.07769 | 0.318183 | 2.034063 | 1.50E-19 | 5.29E-19 |
| AC092718.4 | 2.124274 | 6.032679 | 1.505829 | 2.59E-33 | 2.41E-32 |
| AC108134.3 | 1.340605 | 0.46743 | -1.52006 | 5.58E-45 | 1.17E-43 |
| AL157395.1 | 0.366469 | 0.162108 | -1.17673 | 2.67E-26 | 1.53E-25 |
| AC131011.1 | 0.06167 | 0.148372 | 1.266581 | 0.000187 | 0.000234 |
| ATP2A1-AS1 | 0.313328 | 1.455457 | 2.215727 | 1.13E-43 | 2.10E-42 |
| TTC3-AS1 | 0.077675 | 0.202275 | 1.380792 | 2.78E-09 | 5.15E-09 |
| AC069437.1 | 0.02938 | 0.15854 | 2.431922 | 0.006447 | 0.007008 |
| AC104667.2 | 0.536532 | 1.587863 | 1.56535 | 4.28E-30 | 3.11E-29 |
| AL138789.1 | 0.011144 | 0.110583 | 3.310746 | 4.05E-31 | 3.26E-30 |
| SPON1-AS1 | 0.277877 | 0.11245 | -1.30516 | 1.14E-21 | 4.76E-21 |
| AP000941.1 | 0.51843 | 0.226673 | -1.19354 | 2.10E-31 | 1.82E-30 |
| PCAT6 | 2.375672 | 7.224072 | 1.604477 | 4.74E-36 | 5.09E-35 |
| AC015912.3 | 0.373535 | 1.377005 | 1.88222 | 5.41E-21 | 2.13E-20 |
| AL357568.1 | 0.012584 | 0.196107 | 3.96193 | 0.000267 | 0.000327 |
